# Supplementary material for: The subcortical and neurochemical organization of the ventral and dorsal attention networks
Source: Commun Biol. 2022 Dec 7;5:1343. doi: 10.1038/s42003-022-04281-0 (PMC9729227; doi:10.1038/s42003-022-04281-0)
Supplement: Supplementary file 2 — Supplementary Information [file 42003_2022_4281_MOESM2_ESM.pdf]

Supplementary Information

The subcortical and neurochemical organization of the ventral and dorsal attention networks

Supplementary Table 1. Detailed list of the nodes represented in the VAN connectivity matrices

Group label	Node	Group label	Node	Group label	Node
**Cortical L**	Parcel 23	**Subcortical**	HCaN L	**Cortical R**	Parcel 221
	Parcel 60		Pul L		Parcel 222
	Parcel 61		SC L		Parcel 226
	Parcel 62		PPN/CnF L		Parcel 228
	Parcel 75		Gi L		Parcel 229
	Parcel 79		IPN		Parcel 231
	Parcel 80		MnR		Parcel 237
	Parcel 85		Rpa		Parcel 241
	Parcel 86		HCaN R		Parcel 242
	Parcel 158		Pul R		Parcel 243
	Parcel 161		SC R		Parcel 332
**Cb L**	Cr I L		PPN/CnF R		Parcel 333
	Cr II L		Gi R	**Cb R**	Cr I R
					Cr II R

Nodes are ordered according to their position in the connectivity matrix. Numerated parcel correspond to the cortical parcels of the VAN, described by Gordon and colleagues[1].

Cb, cerebellum; CnF, cuneiform nucleus; Cr I, cerebellar crus I lobule; Cr II, crus II lobule; Gi, gigantocellular nucleus; HCaN, head of caudate nucleus; IPN, interpeduncular nucleus; L, left; MnR, median raphe nucleus; PnO, nucleus pontis oralis; PPN, pedunculopontine nucleus; Pul, pulvinar; R, right; Rpa, raphe pallidus nucleus; SC, superior colliculus.

Supplementary Table 2. Detailed list of the nodes represented in the DAN connectivity matrices

Group label	Node	Group label	Node	Group label	Node
**Cortical L**	Parcel 41	**Subcortical**	HCaN L	**Cortical R**	Parcel 189
	Parcel 42		MD L		Parcel 199
	Parcel 43		Pul L		Parcel 203
	Parcel 49		SC L		Parcel 208
	Parcel 51		PPN/CnF L		Parcel 211
	Parcel 52		Gi L		Parcel 236
	Parcel 55		IPN		Parcel 250
	Parcel 74		Rpa		Parcel 252
	Parcel 87		HCaN R		Parcel 253
	Parcel 88		MD R		Parcel 262
	Parcel 91		Pul R		Parcel 266
	Parcel 92		SC R		Parcel 271
	Parcel 95		PPN/CnF R		Parcel 275
	Parcel 100		Gi R	**Cb R**	HVIIb R
	Parcel 106				HIX R
	Parcel 107				
	Parcel 110				
	Parcel 113				
	Parcel 155				
**Cb L**	Cr I L				
	HVI L				
	HVIIb L				
	HIX L				

Nodes are ordered according to their position in the connectivity matrix. Numerated parcel correspond to the cortical parcels of the DAN, described by Gordon and colleagues[1].

Cb, cerebellum; CnF, cuneiform nucleus; Cr I, cerebellar crus I lobule; Gi, gigantocellular nucleus; HCaN, head of caudate nucleus; HVI, cerebellar lobule VI; HVIIb, cerebellar lobule VIIb; HIX, cerebellar lobule IX; IPN, interpeduncular nucleus; L, left; MD, medial dorsal nucleus of the thalamus; PPN, pedunculopontine nucleus; Pul, pulvinar; R, right; Rpa, raphe pallidus nucleus; SC, superior colliculus.

Supplementary Table 3. Centrality measures of the VAN nodes

Node	Betweenness centrality	Degree centrality	Node	Betweenness centrality	Degree centrality
**Parcel 62**	0.102	0.23	**Cr I R**	0.014	0.28
**Pul R**	0.085	0.26	**Parcel 231**	0.013	0.26
**HCaN L**	0.070	0.28	**Cr II L**	0.012	0.28
**SC L**	0.069	0.28	**Parcel 237**	0.012	0.10
**Parcel 242**	0.067	0.26	**Cr I L**	0.011	0.28
**Parcel 243**	0.051	0.21	**Cr II R**	0.011	0.28
**SC R**	0.050	0.21	**Parcel 333**	0.010	0.23
**Rpa**	0.046	0.31	**Parcel 85**	0.010	0.18
**IPN**	0.045	0.33	**Parcel 222**	0.006	0.23
**Parcel 80**	0.042	0.21	**Parcel 229**	0.005	0.23
**HCaN R**	0.036	0.18	**Gi R**	0.004	0.26
**Parcel 75**	0.036	0.21	**PPN/CnF R**	0.004	0.23
**Pul L**	0.034	0.23	**Parcel 226**	0.003	0.21
**Parcel 241**	0.029	0.18	**Parcel 228**	0.003	0.21
**Parcel 79**	0.028	0.21	**Gi L**	0.000	0.18
**MnR**	0.021	0.31	**Parcel 158**	0.000	0.05
**Parcel 23**	0.020	0.21	**Parcel 161**	0.000	0.10
**Parcel 332**	0.020	0.23	**Parcel 221**	0.000	0.18
**PPN/CnF L**	0.020	0.31	**Parcel 60**	0.000	0.05
**Parcel 86**	0.015	0.18	**Parcel 61**	0.000	0.05

Nodes are ordered according to their betweenness centrality value. Numerated parcel correspond to the cortical parcels of the VAN, described by Gordon and colleagues[1]. CnF, cuneiform nucleus; Cr I, cerebellar crus I lobule; Cr II, cerebellar crus II lobule; Gi, gigantocellular nucleus; HCaN, head of caudate nucleus; IPN, interpeduncular nucleus; L, left; MnR, median raphe nucleus; PnO, nucleus pontis oralis; PPN, pedunculopontine nucleus; Pul, pulvinar; R, right; Rpa, raphe pallidus nucleus; SC, superior colliculus.

Supplementary Table 4. Centrality measures of the DAN nodes

Node	Betweenness centrality	Degree centrality	Node	Betweenness centrality	Degree centrality
**Rpa**	0.056	0.47	**Parcel 74**	0.010	0.22
**Parcel 275**	0.049	0.33	**PPN/CnF L**	0.009	0.31
**Parcel 100**	0.046	0.33	**Parcel 253**	0.009	0.18
**Parcel 87**	0.041	0.35	**HVI L**	0.009	0.27
**Parcel 106**	0.041	0.35	**Parcel 110**	0.009	0.20
**Parcel 41**	0.038	0.35	**Gi L**	0.009	0.29
**MD R**	0.032	0.37	**Gi R**	0.008	0.27
**SC L**	0.031	0.41	**PPN/CnF R**	0.007	0.29
**MD L**	0.030	0.39	**Parcel 42**	0.007	0.22
**Parcel 252**	0.028	0.25	**Parcel 199**	0.007	0.22
**Parcel 189**	0.026	0.31	**HVIIb L**	0.006	0.31
**SC R**	0.025	0.35	**Parcel 107**	0.006	0.20
**Cd L**	0.025	0.35	**Parcel 155**	0.006	0.18
**Cd R**	0.025	0.31	**Parcel 88**	0.005	0.18
**Parcel 49**	0.023	0.31	**HVIIb R**	0.005	0.29
**Cr I L**	0.019	0.33	**Pul L**	0.005	0.22
**Parcel 236**	0.017	0.22	**Parcel 250**	0.004	0.14
**Parcel 51**	0.016	0.24	**Parcel 113**	0.003	0.16
**IPN**	0.015	0.35	**Parcel 52**	0.003	0.14
**Pul R**	0.015	0.27	**Parcel 92**	0.003	0.18
**Parcel 208**	0.014	0.25	**HIX L**	0.002	0.24
**Parcel 91**	0.013	0.27	**Parcel 95**	0.002	0.14
**Parcel 262**	0.013	0.18	**HIX R**	0.002	0.22
**Parcel 43**	0.012	0.24	**Parcel 55**	0.002	0.10
**Parcel 271**	0.011	0.24	**Parcel 211**	0.001	0.10
**Parcel 203**	0.011	0.24	**Parcel 266**	0.000	0.06

Nodes are ordered according to their betweenness centrality value. Numerated parcel correspond to the cortical parcels of the VAN, described by Gordon and colleagues[1]. CnF, cuneiform nucleus; Cr I, cerebellar crus I lobule; Gi, gigantocellular nucleus; HCaN, head of caudate nucleus; HVI, cerebellar lobule VI; HVIIb, cerebellar lobule VIIb; HIX, cerebellar lobule IX; IPN, interpeduncular nucleus; L, left; MD, medial dorsal nucleus of the thalamus; PPN, pedunculopontine nucleus; Pul, pulvinar; R, right; Rpa, raphe pallidus nucleus; SC, superior colliculus.

Supplementary Table 5. Pairwise correlations between neurotransmitter maps and the average VAN structural projection map

Neurotransmitter maps	Spearman's correlation	p-value
A4B2_flubatine_hc30_hillmer	0.53	0.0002
VAChT_feobv_hc5_bedard_sum	0.50	0.0006
VAChT_feobv_hc4_tuominen	0.44	0.0022
DAT_fpcit_hc174_dukart_spect	0.36	0.0206
VAChT_feobv_hc18_aghourian_sum	0.33	0.0218
5HTT_dasb_hc30_savli	0.32	0.0444
5HTT_madam_hc10_fazio	0.30	0.0496
NAT_MRB_hc10_hesse	0.27	0.0326
FDOPA_fluorodopa_hc12_gomez	0.26	0.0532
5HTT_dasb_hc100_beliveau	0.24	0.1458
D2_flb457_hc37_smith	0.17	0.3723
NAT_MRB_hc77_ding	0.16	0.1558
D2_flb457_hc55_sandiego	0.16	0.4155
D2_raclopride_hc7_alakurtti	0.15	0.3879
D2_fallypride_hc49_jaworska	0.14	0.4463
DAT_fepe2i_hc6_sasaki	0.14	0.4195
5HT4_sb20_hc59_beliveau	-0.04	0.8392
MU_carfentanil_hc204_kantonen	-0.04	0.8364
MU_carfentanil_hc39_turtonen	-0.14	0.5565
D1_SCH23390_hc13_kaller	-0.19	0.2565
H3_cban_hc8_gallezot	-0.22	0.3431
5HT1b_az_hc36_beliveau	-0.40	0.0020
5HT1b_p943_hc65_gallezot	-0.44	0.0002
5HT1b_p943_hc22_savli	-0.59	0.0002
CB1_omar_hc77_normandin	-0.59	0.0002
5HT6_gsk_hc30_radnakrishnan	-0.59	0.0002
GABAa-bz_flumazenil_hc16_norgaard	-0.60	0.0002
mGluR5_abp_hc22_rosaneto	-0.62	0.0002
M1_lsn_hc24_naganawa	-0.69	0.0002
5HT1a_cumi_hc8_beliveau	-0.70	0.0002
CB1_FMPEPd2_hc22_laurikainen	-0.73	0.0002
5HT1a_way_hc36_savli	-0.74	0.0002
mGluR5_abp_hc73_smart	-0.75	0.0002
mGluR5_abp_hc28_dubois	-0.75	0.0002
GABAa_flumazenil_hc6_dukart	-0.75	0.0002
5HT2a_mdl_hc3_talbot	-0.78	0.0002
5HT2a_cimbi_hc29_beliveau	-0.78	0.0002
5HT2a_alt_hc19_savli	-0.80	0.0002

The names of neurotransmitter maps denote the neurotransmitter system abbreviation followed by the original work they were derived, according to Hansen and colleagues[2]. 5HT1a, serotonin 1a receptors; 5HT1b, serotonin 1b receptors; 5HT2a, serotonin 2a receptors; 5HTT, serotonin transporters; A4B2, acetylcholine $\alpha 4\beta 2$ nicotinic receptors; CB1, cannabinoid receptors 1; D1, dopamine receptors 1; D2, dopamine receptors 2; DAT, dopamine transporters; FDOPA, fluorodopa; GABAa, GABAa receptors; H3, histamine receptors 3; M1, muscarinic receptors 1; mGluR5, metabotropic glutamate receptors 5; MU, mu-opioid receptors; NAT, noradrenaline transporters; VAchT, vesicular acetylcholine transporters.

Supplementary Table 6. Pairwise correlations between neurotransmitter maps and the average DAN structural projection map.

Neurotransmitter maps	Spearman's correlation	p-value
A4B2_flubatine_hc30_hillmer	0.59	0.0002
VAChT_feobv_hc5_bedard_sum	0.55	0.0004
VAChT_feobv_hc4_tuominen	0.50	0.0008
DAT_fpcit_hc174_dukart_spect	0.41	0.0306
VAChT_feobv_hc18_aghourian_sum	0.40	0.0202
5HTT_dasb_hc30_savli	0.37	0.0582
5HTT_madam_hc10_fazio	0.36	0.0586
NAT_MRB_hc10_hesse	0.33	0.0314
5HTT_dasb_hc100_beliveau	0.29	0.1572
FDOPA_fluorodopa_hc12_gomez	0.28	0.0722
D2_flb457_hc37_smith	0.22	0.3655
NAT_MRB_hc77_ding	0.21	0.1150
D2_flb457_hc55_sandiego	0.21	0.4007
D2_fallypride_hc49_jaworska	0.19	0.4353
D2_raclopride_hc7_alakurtti	0.19	0.3827
DAT_fepe2i_hc6_sasaki	0.17	0.4563
MU_carfentanil_hc204_kantonen	0.01	0.9712
5HT4_sb20_hc59_beliveau	0.00	0.9858
MU_carfentanil_hc39_turtonen	-0.08	0.7984
H3_cban_hc8_gallezot	-0.16	0.6165
D1_SCH23390_hc13_kaller	-0.16	0.5101
5HT1b_az_hc36_beliveau	-0.39	0.0160
5HT1b_p943_hc65_gallezot	-0.43	0.0030
5HT1b_p943_hc22_savli	-0.59	0.0002
5HT6_gsk_hc30_radnakrishnan	-0.59	0.0002
mGluR5_abp_hc22_rosaneto	-0.61	0.0002
CB1_omar_hc77_normandin	-0.62	0.0002
GABAa-bz_flumazenil_hc16_norgaard	-0.62	0.0002
5HT1a_cumi_hc8_beliveau	-0.69	0.0002
M1_lsn_hc24_naganawa	-0.70	0.0002
mGluR5_abp_hc28_dubois	-0.74	0.0002
CB1_FMPEPd2_hc22_laurikainen	-0.74	0.0002
mGluR5_abp_hc73_smart	-0.75	0.0002
5HT1a_way_hc36_savli	-0.76	0.0002
GABAa_flumazenil_hc6_dukart	-0.78	0.0002
5HT2a_cimbi_hc29_beliveau	-0.79	0.0002
5HT2a_alt_hc19_savli	-0.82	0.0002
5HT2a_mdl_hc3_talbot	-0.82	0.0002

The names of neurotransmitter maps denote the neurotransmitter system abbreviation followed by the original work they were derived, according to Hansen and colleagues[2]. 5HT1a, serotonin 1a receptors; 5HT1b, serotonin 1b receptors; 5HT2a, serotonin 2a receptors; 5HTT, serotonin transporters; A4B2, acetylcholine $\alpha4\beta2$ nicotinic receptors; CB1, cannabinoid receptors 1; D1, dopamine receptors 1; D2, dopamine receptors 2; DAT, dopamine transporters; FDOPA, fluorodopa; GABAa, GABAa receptors; H3, histamine receptors 3; M1, muscarinic receptors 1; mGluR5, metabotropic glutamate receptors 5; MU, mu-opioid receptors; NAT, noradrenaline transporters; VAchT, vesicular acetylcholine transporters.

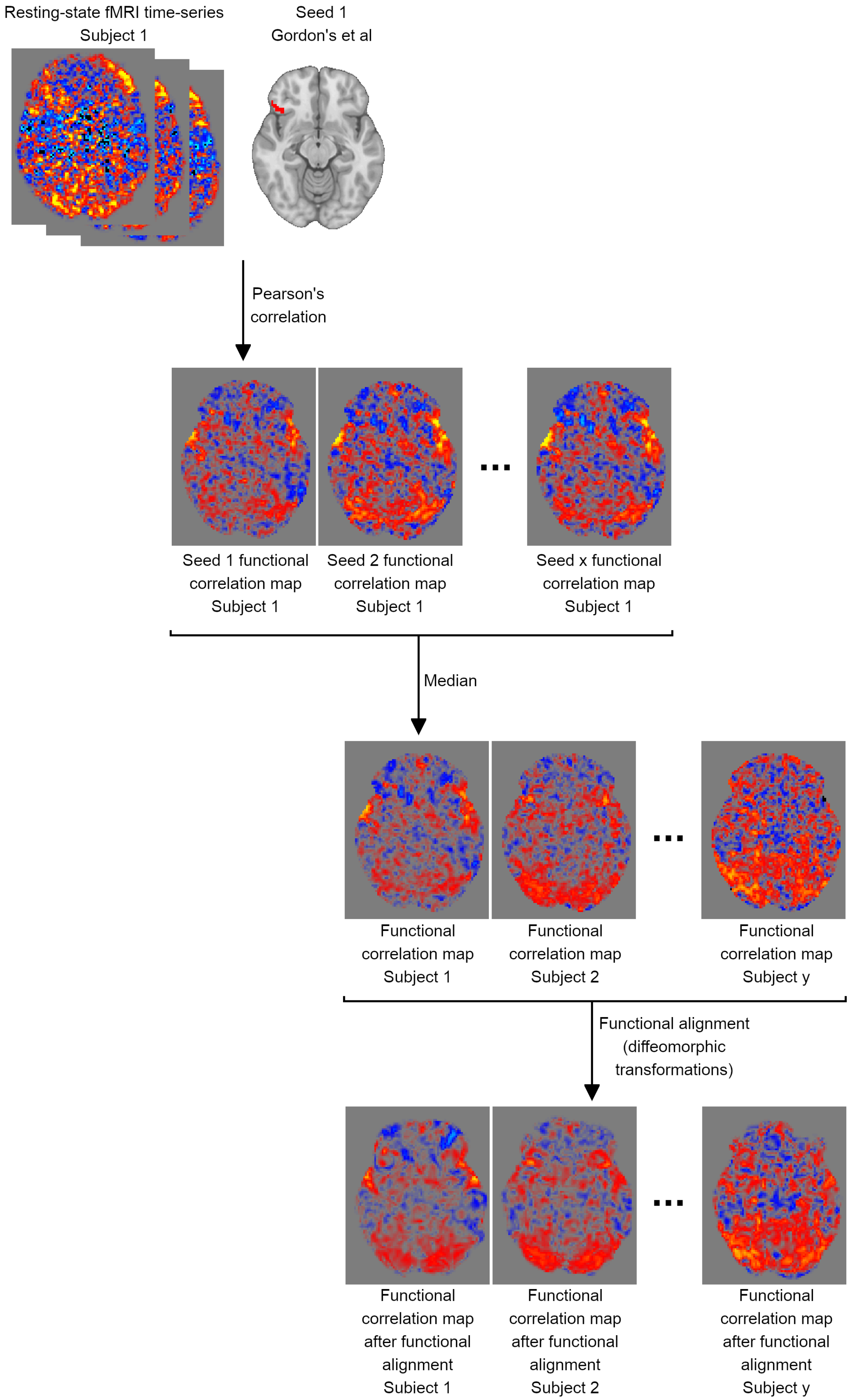
Resting-state fMRI time-series
Subject 1
Seed 1
Gordon's et al
Pearson's
correlation
Seed 1 functional
correlation map
Subject 1
Seed 2 functional
correlation map
Subject 1
Seed x functional
correlation map
Subject 1
Median
Functional
correlation map
Subject 1
Functional
correlation map
Subject 2
Functional
correlation map
Subject y
Functional alignment
(diffeomorphic
transformations)
Functional
correlation map
after functional
alignment
Subject 1
Functional
correlation map
after functional
alignment
Subject 2
Functional
correlation map
after functional
alignment
Subject y

Supplementary Figure 1. Schematic representation of the steps performed during functional alignment. First. seed-based resting-state functional correlation maps were computed (Pearson's correlation). Second. the median of the Pearson's correlation maps was calculated to obtain the subjects' functional correlation map of the studied network (VAN or DAN). Third. the subjects' functional correlation maps were functionally aligned using diffeomorphic transformations.

Supplementary References

1. Gordon EM, Laumann TO, Adeyemo B, et al (2016) Generation and Evaluation of a Cortical Area Parcellation from Resting-State Correlations. Cereb Cortex 26:288–303. https://doi.org/10.1093/cercor/bhu239
2. Hansen JY, Shafiei G, Markello RD, et al (2021) Mapping neurotransmitter systems to the structural and functional organization of the human neocortex. bioRxiv 2021.10.28.466336. https://doi.org/10.1101/2021.10.28.466336
