# Supplementary material for: The subcortical and neurochemical organization of the ventral and dorsal attention networks
Source: Commun Biol. 2022 Dec 7;5:1343. doi: 10.1038/s42003-022-04281-0 (PMC9729227; doi:10.1038/s42003-022-04281-0)
Supplement: Supplementary file 3 — Description of Additional Supplementary Data [file 42003_2022_4281_MOESM3_ESM.docx]

**Description of Additional Supplementary Files**

**File name:** Supplementary Data 1

**Description:** The source data behind figure 4.

**File name:** Supplementary Data 2

**Description:** The source data behind figures 6a and 6c.
