# Supplementary material for: The subcortical and neurochemical organization of the ventral and dorsal attention networks
Source: Commun Biol. 2022 Dec 7;5:1343. doi: 10.1038/s42003-022-04281-0 (PMC9729227; doi:10.1038/s42003-022-04281-0)
Supplement: Supplementary file 6 — Reporting Summary [file 42003_2022_4281_MOESM6_ESM.pdf]

nature portfolio

Corresponding author(s): Pedro Nascimento Alves
Michel Thiebaut de Schotten

Last updated by author(s): Oct 11, 2022

Reporting Summary

Nature Portfolio wishes to improve the reproducibility of the work that we publish. This form provides structure for consistency and transparency in reporting. For further information on Nature Portfolio policies, see our Editorial Policies and the Editorial Policy Checklist.

Statistics

For all statistical analyses, confirm that the following items are present in the figure legend, table legend, main text, or Methods section.

n/a	Confirmed	
☐	☒	The exact sample size (*n*) for each experimental group/condition, given as a discrete number and unit of measurement
☒	☐	A statement on whether measurements were taken from distinct samples or whether the same sample was measured repeatedly
☐	☒	The statistical test(s) used AND whether they are one- or two-sided<br>*Only common tests should be described solely by name; describe more complex techniques in the Methods section.*
☐	☒	A description of all covariates tested
☐	☒	A description of any assumptions or corrections, such as tests of normality and adjustment for multiple comparisons
☐	☒	A full description of the statistical parameters including central tendency (e.g. means) or other basic estimates (e.g. regression coefficient) AND variation (e.g. standard deviation) or associated estimates of uncertainty (e.g. confidence intervals)
☐	☒	For null hypothesis testing, the test statistic (e.g. *F*, *t*, *r*) with confidence intervals, effect sizes, degrees of freedom and *P* value noted<br>*Give P values as exact values whenever suitable.*
☒	☐	For Bayesian analysis, information on the choice of priors and Markov chain Monte Carlo settings
☒	☐	For hierarchical and complex designs, identification of the appropriate level for tests and full reporting of outcomes
☐	☒	Estimates of effect sizes (e.g. Cohen's *d*, Pearson's *r*), indicating how they were calculated

Our web collection on statistics for biologists contains articles on many of the points above.

Software and code

Policy information about availability of computer code

Data collection	N/A
Data analysis	We used our homemade open software: bcbtoolkit (http://toolkit.bcblab.com)

For manuscripts utilizing custom algorithms or software that are central to the research but not yet described in published literature, software must be made available to editors and reviewers. We strongly encourage code deposition in a community repository (e.g. GitHub). See the Nature Portfolio guidelines for submitting code & software for further information.

Data

Policy information about availability of data

All manuscripts must include a data availability statement. This statement should provide the following information, where applicable:

- Accession codes, unique identifiers, or web links for publicly available datasets
- A description of any restrictions on data availability
- For clinical datasets or third party data, please ensure that the statement adheres to our policy

Reported maps are openly available at https://neurovault.org/collections/XONZLGPJ/.

Field-specific reporting

Please select the one below that is the best fit for your research. If you are not sure, read the appropriate sections before making your selection.

☒ Life sciences ☐ Behavioural & social sciences ☐ Ecological, evolutionary & environmental sciences

For a reference copy of the document with all sections, see nature.com/documents/nr-reporting-summary-flat.pdf

Life sciences study design

All studies must disclose on these points even when the disclosure is negative.

Sample size	110 participants for functional connectivity analysis and 177 for structural connectivity analysis. No sample size calculation was performed. These sample sizes are standard when describing main neuroimaging effects.
Data exclusions	No data exclusions.
Replication	The functional connectivity findings were replicated in the structural connectivity and neurochemical analyses.
Randomization	N/A
Blinding	N/A

Reporting for specific materials, systems and methods

We require information from authors about some types of materials, experimental systems and methods used in many studies. Here, indicate whether each material, system or method listed is relevant to your study. If you are not sure if a list item applies to your research, read the appropriate section before selecting a response.

Materials & experimental systems

n/a	Involved in the study	
☒	☐	Antibodies
☒	☐	Eukaryotic cell lines
☒	☐	Palaeontology and archaeology
☒	☐	Animals and other organisms
☒	☐	Human research participants
☒	☐	Clinical data
☒	☐	Dual use research of concern

Methods

n/a	Involved in the study	
☒	☐	ChIP-seq
☒	☐	Flow cytometry
☐	☒	MRI-based neuroimaging

Magnetic resonance imaging

Experimental design

Design type	Resting-state
Design specifications	N/A
Behavioral performance measures	NA

Acquisition

Imaging type(s)	structural - functional - diffusion
Field strength	7T
Sequence & imaging parameters	Detailed in the Human Connectome Project protocol (http://www.humanconnectome.org/storage/app/media/documentation/s1200/HCP_S1200_Release_Reference_Manual.pdf; Glasser et al. 2013).
Area of acquisition	Brain

Diffusion MRI ☒ Used ☐ Not used

Parameters: Detailed in the Human Connectome Project protocol (http://www.humanconnectome.org/storage/app/media/documentation/

Parameters | s1200/HCP_S1200_Release_Reference_Manual.pdf; Glasser et al. 2013).

Preprocessing

Preprocessing software	BCBtoolkit
Normalization	Diffeormorphic / functional
Normalization template	ICBM152
Noise and artifact removal	Detailed in the Human Connectome Project protocol (http://www.humanconnectome.org/storage/app/media/documentation/s1200/HCP_S1200_Release_Reference_Manual.pdf; Glasser et al. 2013).
Volume censoring	N/A

Statistical modeling & inference

Model type and settings	Pearson correlation and t-tests
Effect(s) tested	ATLASES

Specify type of analysis: ☐ Whole brain ☐ ROI-based ☒ Both

Anatomical location(s): previously published atlases

Statistic type for inference (See Eklund et al. 2016)	voxel-wise
Correction	permutation

Models & analysis

n/a	Involved in the study
☐	☒ Functional and/or effective connectivity
☐	☒ Graph analysis
☒	☐ Multivariate modeling or predictive analysis

Functional and/or effective connectivity	Pearson correlation and partial correlation
Graph analysis	Binarized graphs, based on structural connectivity. Group and subject-level betweenness centrality and degree centrality were computed.
